# Modulations of EEG Beta Power during Planning and Execution of Grasping Movements

**DOI:** 10.1371/journal.pone.0060060

**Published:** 2013-03-21

**Authors:** Manuel Zaepffel, Romain Trachel, Bjørg Elisabeth Kilavik, Thomas Brochier

**Affiliations:** 1 Institut de Neurosciences Timone, UMR 7289, CNRS, Aix-Marseille Université, Marseille, France; 2 INRIA, Sophia-Antipolis Méditerranée, Athena Project-Team, Sophia-Antipolis, France; University of Rome, Italy

## Abstract

Although beta oscillations (≈ 13–35 Hz) are often considered as a sensorimotor rhythm, their functional role remains debated. In particular, the modulations of beta power during preparation and execution of complex movements in different contexts were barely investigated. Here, we analysed the beta oscillations recorded with electroencephalography (EEG) in a precued grasping task in which we manipulated two critical parameters: the grip type (precision *vs.* side grip) and the force (high *vs.* low force) required to pull an object along a horizontal axis. A cue was presented 3 s before a GO signal and provided full, partial or no information about the two movement parameters. We measured beta power over the centro-parietal areas during movement preparation and execution as well as during object hold. We explored the modulations of power in relation to the amount and type of prior information provided by the cue. We also investigated how beta power was affected by the grip and force parameters.

We observed an increase in beta power around the cue onset followed by a decrease during movement preparation and execution. These modulations were followed by a transient power increase during object hold. This pattern of modulations did not differ between the 4 movement types (2 grips ×2 forces). However, the amount and type of prior information provided by the cue had a significant effect on the beta power during the preparatory delay. We discuss how these results fit with current hypotheses on the functional role of beta oscillations.

## Introduction

More than 50 years ago, it was shown that the power of brain oscillations in the beta-band (≈13–35 Hz) is modulated in relation to voluntary movements [1]. When compared to a control state an increase in power is often referred to as an event-related synchronization (ERS), whereas a decrease is referred to as an event-related desychronization (ERD) [2]. Typically, a voluntary movement is preceded by a beta ERD, with power decreasing gradually to reach a minimum during movement execution. This ERD is followed by a phasic ERS after movement termination known as the “beta rebound”. Such changes in oscillatory activity are reported in a large number of studies using different motor tasks and recording techniques [3], [4], [5], [6], [7], [8], [9], [10], [11], [12]. They are found in many cerebral structures [8], but mainly around the sensorimotor cortex, with a contralateral predominance [13], [14]. The ERD/ERS pattern is altered in patients with motor impairments [15] and both the oscillatory activity and motor performance can be restored in Parkinson's patients by means of L-dopa treatment or stimulation of the subthalamic nucleus [16].

Although the link between the beta rhythm and motor control is now well established, the relevance and functional significance of this rhythm is still debated [17]. Recently, Jenkinson and Brown [18] proposed that the cortical beta activity is characterized by a “functional polymorphism”. This hypothesis suggests that cortical beta rhythm may originate from several sources and reflect distinct functional processes [19], [20], [21]. In line with this assumption, it is known that many experimental factors can affect the sensorimotor beta rhythm. For instance, it has been shown that the ERD during movement preparation is modulated by the uncertainty about the direction of a forthcoming movement [22]. The amplitude of the beta activity also varies with temporal attention; it peaks around the onset of relevant cues instructing the subject about the direction of a forthcoming movement [23]. Others suggest that the beta oscillations reflect the “status quo”, i.e. the maintenance of the current sensorimotor or cognitive state [17]. Finally, the typical ERD/ERS pattern is also observed during motor imagery [10], [12], [24], passive movement [25] and action observation [26].

Altogether, these studies suggest that the beta oscillations are related to several cognitive aspects of motor control. Furthermore, other experiments showed that beta oscillations are linked to the control of specific movement parameters, such as the type of grip for the grasping of differently shaped objects [27] and the force used to lift a weight [28]. This assumption is challenged by studies showing that oscillations in the beta range are little informative to decode movement parameters such as the direction of reaching movements [29] or the grip type and force to grasp an object [30]. Therefore, the modulation of beta oscillations with motor parameters remains controversial. One source of this controversy may relate to the fact that beta modulations in these different studies were analyzed in different time windows, before, during or after movement execution.

Given this background, the main objective of the present study is to analyze in a single experiment the modulations of beta power during preparation and execution of grasping movements while manipulating two movement parameters in different contexts. For this purpose, we use a pre-cueing paradigm in which a GO signal is preceded by a cue providing partial, full or no information about two parameters critical for grasping: the grip type to grasp an object and the overall force required for pulling it.

The particularity of this pre-cueing task is that it allows investigating the neurophysiological correlates related to the processing of one parameter alone or in combination with another. The analysis of beta modulations in such an experimental context should improve our understanding of the functional role of beta oscillations, as well as the functional organization of the motor system. The latter is commonly addressed with a similar approach based on the analysis of event-related potentials (ERPs) and reaction time (RT) [31], [32]. Moreover, following to the “functional polymorphism” hypothesis [18], the pre-cueing conditions and the motor parameters may affect differently the modulation of beta power in one or several epochs of the task. These differences can then be directly confronted with current hypotheses about the functional roles of beta oscillations.

## Methods

### Participants

14 right-handed subjects voluntarily participated in the experiment (5 men, mean age = 24 years, age range = 21 to 41 years). They all had normal or corrected-to-normal vision and no medical history that might interfere with the task. The study was approved by the local ethics committee of the Aix-Marseille University. All subjects gave written informed consent prior to participation.

### Experimental task

**Figure 1:**
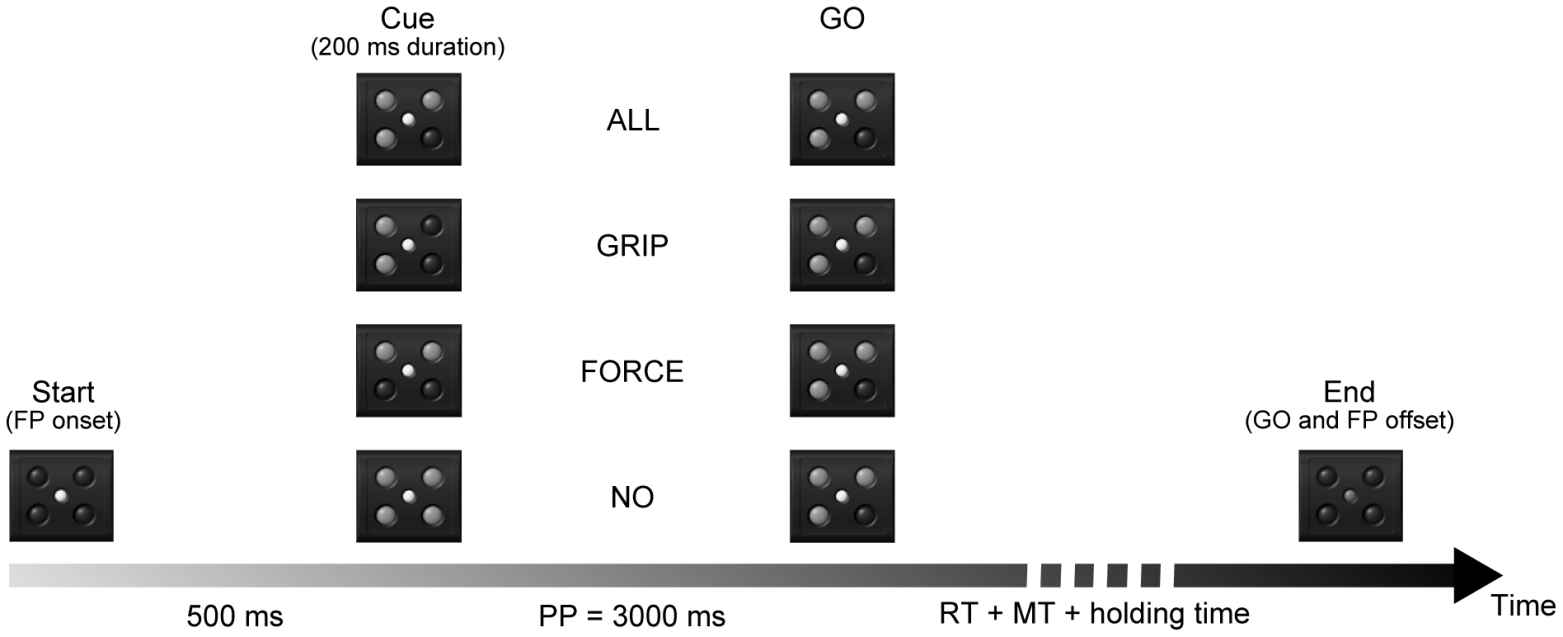
The sequence of events for one typical trial. The closures of two switches initiate the trial and trigger the illumination of the fixation point (FP). In this example, a side grip (two left LEDs) is required to reach, grasp and pull a heavy (two top LEDs) object. For each pre-cueing condition (ALL, GRIP, FORCE, NO), presented in separate block, the GO systematically provides the combined information about the required grip and force. When the object has been held for 1 s, the GO and FP extinction indicates the end of the trial.

The goal of the task was to use one of two different grip types to reach, grasp and pull an object that could be either heavy or light. Subjects sat in an adjustable chair in front of the experimental apparatus at comfortable distance and height [33]. Two switches were used to initiate the trials. The target object was a parallelepiped (60×38×30 mm) rotated 45° from the vertical axis. It was located 13 cm behind and 14 cm above the switches. A CRT monitor (17 in.) was placed behind the apparatus at 1 m viewing distance. The object and the monitor were aligned with the midsagittal plane. Visual cues were made up of 5 large LED-like signals. Four red LEDs (1,2° visual angle) were positioned in a square (4° visual angle). In the middle of this square a yellow LED (0.8° visual angle) was used as an eye fixation point (FP). The 4 peripheral red LEDs were used for the cue and the GO. The illumination of the two right LEDs instructed the subject to use a precision grip (PG) to grasp the object between the tips of the index finger and thumb. The two left LEDs instructed the subject to use a side grip (SG) between the tip of the thumb and the lateral surface of the index finger. Illumination of the two bottom or top LEDs instructed the subject that pulling the object required a low force (LF, object weight = 200 g) or high force (HF, object weight = 700 g), respectively. The grip and force cues could be combined to instruct the subject to perform one of the four possible grasps (see Figure 1).

Every trial followed the same sequence of events (see Figure 1). Subjects self-initiated each trial at their own pace by positioning their hands on the two switches. Switches closure triggered FP illumination. 500 ms after FP onset the pre-cue was illuminated for 200 ms. The preparatory period (PP) between the pre-cue onset and the imperative GO signal lasted 3 s. The GO signal instructed the subjects to reach, grasp and pull the object using the right hand while the left hand had to remain on the left switch. Following the object displacement, the subject had to hold the object in a stable position for 1 s. At the end of this holding period, extinction of all signals (GO and FP) indicated the end of the trial. Subjects were instructed to react as fast as possible to the GO signal and to avoid impeding movements such as eye blinks, saccades, left hand movements or leg movements throughout the trial. They were also constrained to keep the object displacement velocity within a narrow range (90 mm/s<velocity peak<160 mm/s) for both the high and low weights. The reaction time (RT) and movement time (MT) had to be below 700 ms and 400 ms, respectively. The trial was aborted and an error feedback was displayed when the subject's performance failed to match the task requirements.

Since we used a fixed PP duration (3 s) the subjects could anticipate the GO signal. To prevent subjects anticipating the GO onset, trials in which the RT was below 150 ms were also aborted. In addition, no-go trials were randomly presented. No-go trials were similar to other trials except that the GO did not appear. The subjects had to keep their hands on the switches and wait until a positive feedback appeared on the screen (“NOGO réussi” i.e. successful no-go) which indicated the end of the trial.

We used four experimental pre-cueing conditions in which the cue provided full, partial or no prior information about the movement parameters (see Figure 1). In the four conditions, the GO always provided all the information about grip and force.

In the “ALL” condition, the cue provided information about both grip and force (4 different cues, PGHF, PGLF, SGHF, SGLF).

In the “GRIP” condition, the cue provided information only about the grip (2 different cues, SG or PG).

In the “FORCE” condition, the cue provided information only about the force (2 different cues, HF or LF).

In the “NO” condition, the cue provided no information (all four red LEDs illuminated). The subject had to wait until the GO to know which movement to perform.

The experiment was divided into two sessions: a training session and an experimental session the following day. Before the experimental session on the 2^nd^ day, the subject first performed another training set (4 blocks of 10 trials, one block per pre-cueing condition) before being prepared for EEG recordings. The experimental session was divided into 8 blocks of trials (2 per pre-cueing condition) presented in a pseudo-random order. Within each block, the subject had to perform 44 correct trials presented in a random order, 10 for each response type (PGHF, PGLF, SGHF or PGLF) and 4 no-go (10%). All failed trials were reintegrated and presented randomly later in the block. Each subjects performed a total of 352 correct trials (88 per pre-cueing conditions) during the experimental session.

### Data recordings and analysis

#### Behavioral data

A custom-written software in Labview 8.5 (National Instruments) was used to control the task and to measure RT, MT and errors. The RT was defined as the time between the GO onset and the right switch release. The MT was defined as the time between the switch release and object contact (i.e. the reaching time). The grip force, load force and object horizontal displacement were also recorded. The behavioral results have been described elsewhere [33]. The behavioral findings relevant to the present study are briefly summarized in the results.

#### Electrophysiological data

The EEG was recorded from 62 Ag/AgCl electrodes mounted on an elastic cap (“Waveguard Active Shield”, ANT) positioned according to the 10–20 method. The different EEG electrodes had a common, average reference. Skin-electrode impedances were kept below 10 kΩ. The EEG on each electrode was amplified using a Refa8 “High-density” full band DC amplifier (Advanced Neuro Technology, ANT) providing default low-pass filtering of the signal at 0.27*sampling rate. No additional filtering was used during the recordings. Bipolar electrodes were used to record the electrooculogram (EOG). All signals were sampled at 1024 Hz with ASA 4.0 (ANT Software). Incorrect trials and correct trials with visually identified EOG artefacts (blink or saccade) were excluded from the analyses. Similarly, we excluded correct trials showing slow drifts in the EEG signal on any electrodes(>100 µV).

To obtain reference-free data and to emphasize the local features of the artefact-free EEG signals, we first computed the current sources density estimation (CSD, i.e. surface laplacian) using the CSD toolbox [34], which is based on the spherical spline algorithm [35]. CSD data were used for all subsequent analyses. Each trial corresponded to a 7600 ms time-window time-locked to the GO signal, starting 1000 ms before FP onset and ending after the end of the holding period.

**Figure 2:**
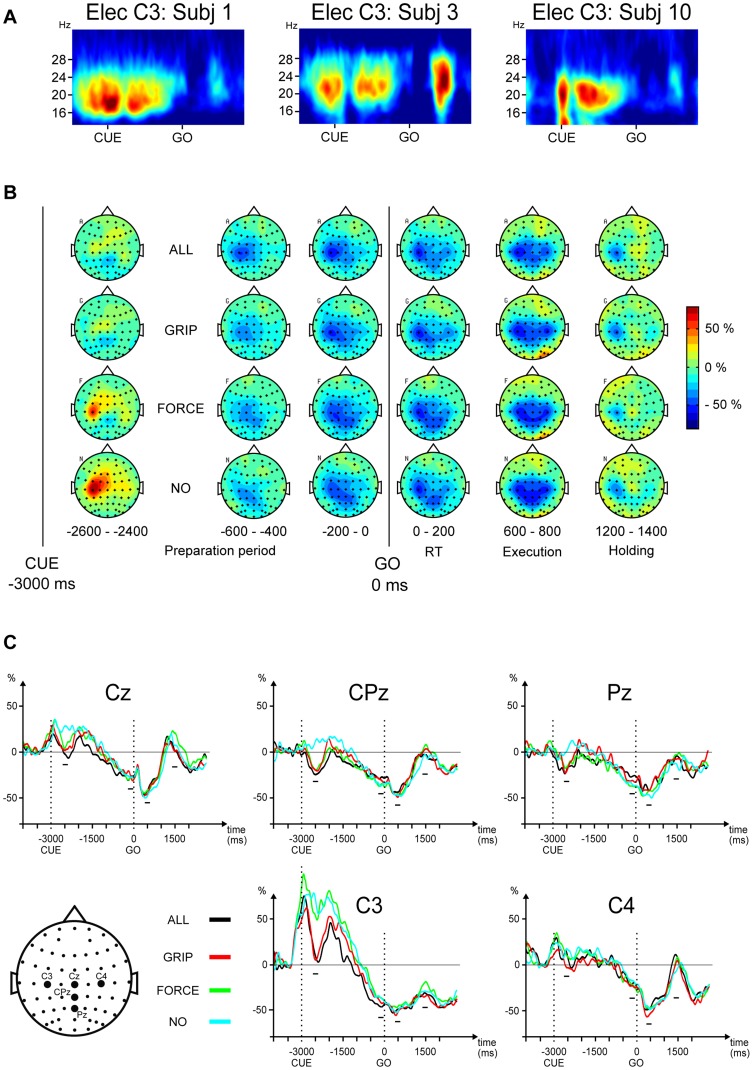
A: Time-frequency maps averaged across all conditions for 3 typical subjects (electrode C3). For all the subjects, the modulations in beta power were maximal in the 15–25 Hz frequency band. **B**: Topographic maps (grand average) of the ERD/ERS modulations expressed in percentages compared to the baseline (0 %) as a function of pre-cueing conditions. Each map represents the averaged signal for a 200 ms time window. **C**: Averaged ERD/ERS traces as a function of pre-cueing conditions for the 5 electrodes of interest (Cz, CPz, Pz, C3, C4). The two vertical dotted lines represent the cue and GO onsets respectively. The 4 short horizontal bars represent the 4 time windows used for statistical analyses.

We used the OpenElectrophy toolbox [36] to compute time-frequency (TF) maps. This computation was performed with continuous wavelet transform [37], using a Morlet wavelet. Visual inspection of TF maps within the 13–35 Hz β-band revealed strong modulations of beta power around 20 Hz for all the 14 subjects (Figure 2a). To assess whether these modulations differed between the pre-cueing conditions and the response types, we computed the ERD/ERS using the “band power method” [2] within a 15–25 Hz frequency window for all the subjects. For each subject, the signals from each trial were first filtered between 15–25 Hz using a Butterworth band-pass filter (order = 5) before being squared, averaged for each pre-cueing condition and response type separately and then smoothed with a gaussian convolution (200 ms width). Finally, the baseline (200 ms window after FP onset) was subtracted and the data converted into percentages by dividing the result of this subtraction by baseline*100.

### Statistical analysis

**Figure 3:**
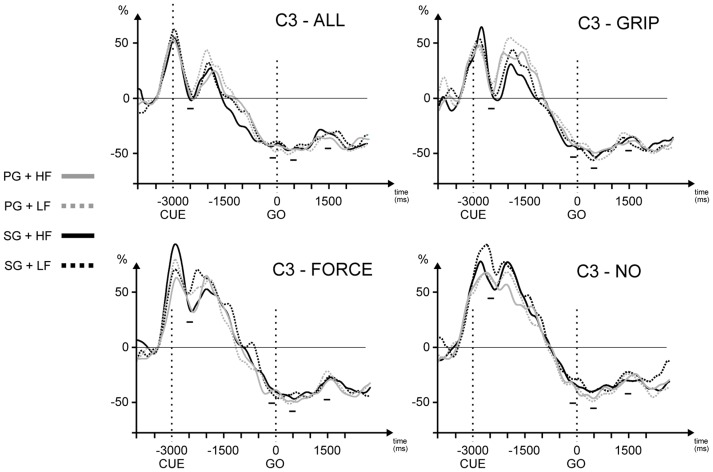
ERD/ERS traces as a function of behavioral responses for the electrode C3 and each pre-cueing condition. The two vertical dotted lines represent cue and GO onsets respectively. PG + HF: precision grip and high force, PG + LF: precision grip and low force, SG + HF: side grip and high force, SG + LF: side grip and low force. The 4 short horizontal bars represent the 4 time windows used for statistical analyses. For visual purposes, data in the figure were Gaussian-convolved with a 500 ms window.

Based on the ERD/ERS topographic maps (Figure 2b), we selected 5 electrodes of interest (Cz, CPz, Pz, C3, C4) covering the centro-parietal zone where the modulations of the beta power were maximal. These electrodes are commonly analyzed in motor control studies since they are located above cortical regions known to be involved in movement preparation and execution [38], [39]. To perform the statistical analyses, we also selected 4 time windows (200 ms width) corresponding to 4 specific stages in the task:

- The “Early-PP” window corresponded to the initial part of the PP, shortly after the cue presentation. In this epoch the pre-cueing conditions were maximally differentiated. The latency of this maximal difference, similar for all the electrodes, was approximately 500 ms after the cue onset (see Figure 2c). This latency also corresponded to a transient trough in beta power reported in other studies [40], [41]. Thus, a 400–600 ms time window was used for statistical analyses (Figure 2c).
- The “Late-PP” window corresponded to the late part of the PP preceding the GO (figure 2c). We selected this time window to specifically compare the ERD/ERS modulation with the modulation of slow ERPs occurring during the PP. In particular, the late part of the contingent negative variation (late CNV) was typically analyzed in a 200 ms interval immediately preceding the GO and its modulation with pre-cueing conditions was used to assess the functional organization of motor planning processes [31], [33].
- The “EXEC” window corresponded to the dynamic phase of movement execution occurring approximately 500 ms after the GO signal. This period also corresponded to the time of maximal ERD amplitude (see Figure 2). The 200 ms time window used for statistical analyses was taken from 400 ms to 600 ms after GO onset.
- The “HOLD” window corresponded to the object holding period occurring approximately 1500 ms after the GO signal. This window was selected based on the peak amplitude of the rebound following the movement-related ERD (see Figure 2). As shown on the time-frequency maps (Figure 3a), the latency of the beta rebound was very similar between subjects. Therefore, the 200 ms time window used for statistical analyses was taken from 1400 ms to 1600 ms after GO onset.

A 5-way repeated measures ANOVA was used to analyze ERD/ERS amplitude with the 5 electrodes (Cz, CPz, Pz, C3, C4), the 4 time windows (Early-PP, Late-PP, EXEC, HOLD), the 4 precuing conditions (ALL, GRIP, FORCE, NO), the 2 grip types (PG, SG) and finally the 2 force (HF, LF) as within-subject factors. Since the electrodes × pre-cueing condition × time window interaction was significant (see Results), we investigated the topographic differences in detail by additionally performing, on each electrode and each time window individually, a 3-way repeated measures ANOVA with pre-cueing conditions, grip type, and force as within-subject factors. This approach was subject to a Type I error rate inflation; increasing the number of tests increases the risk of observing a false-positive [42]. We therefore corrected the .05 α threshold using the Bonferroni adjustment in which .05 α threshold was divided by the number of tests performed. For all the ANOVAs, when the sphericity assumption was not met, we used a corrected *p* value [43] and reported the corresponding *ε* value. Post hoc procedures were performed with Tukey's test.

## Results

### Behavioral results

Behavioral results were described in detail elsewhere [33]. Importantly, they showed that the subjects followed the task requirements by using the cue to react as fast as possible to the GO signal. Indeed, RT decreased with the amount of prior information. While RT was affected by the pre-cueing conditions, MT was only affected by the response types (faster movements for HF). These effects indicated that the pre-cueing selectively affects processes preceding movement execution. They also confirmed that the force was planned in advance since the MT and force profiles were typical of anticipatory force adjustments.

### Electrophysiological results

Since the pre-cueing conditions were presented in separate blocks of trials, we first controlled that the beta power during the baseline period was not modulated by anticipatory processes related to the expected content of the cue. It is conceivable that subjects did not anticipate the cue in the same way when it contained no information or when it could be used for movement preparation. We therefore compared, before baseline subtraction, the average baseline beta power between the 4 pre-cueing conditions. We used a 4-way repeated measures ANOVA with electrode, pre-cueing condition, grip and force as within-subject factors. We found no significant main effect or interactions related to the pre-cueing conditions (all *p*-values>.1). Thus, the block design for the presentation of pre-cuing conditions did not affect the baseline beta power significantly. The following analyzes were therefore done on data with the baseline subtracted (i.e. ERD/ERS).

The 5-way ANOVA (factors: electrode, time window, pre-cueing, grip type, force) used to analyze ERD/ERS modulations with the different experimental factors revealed 2 significant main effects and 4 significant interactions. No other effects were significant (all *p*-values >.2).

The first main effect was for the time windows (*F*(3,39) = 26.611, *p*<.001), meaning that the average ERD/ERS amplitude varied between at least 2 of the 4 time windows as already suggested by the individual TF maps (see Figure 2a). These maps show a relatively high magnitude of β power (at about 20 Hz) during the first part of the PP (Early-PP), which contrast with the lowest magnitude observed during the dynamic phase of movement execution (EXEC). Below, the power increase during the first part of the PP is referred to as “ERS_cue_” while the minimal power during execution is referred to as “ERD”.

Furthermore, the time window × electrode interaction (*F*(12,156) = 8.162, *p*<.001) refines the main effect of time windows. This interaction shows that the magnitude of the ERS_cue_ during Early-PP was stronger on C3 than on CPz, where it was almost absent. During EXEC, the ERD magnitude was similar across electrodes (see Figure 2c). In addition, during the HOLD, an ERS was observed on Cz while the average power observed on C3 remained below the baseline level.

The remaining significant results of the ANOVA reveal that the different pre-cueing conditions had an effect on the ERD/ERS amplitude and that this effect varied between the selected time windows and electrodes (see Figure 2c). These effects were: (1) the main effect of pre-cueing condition (*F*(3,39) = 2.868, *p* = .046), (2) the pre-cueing condition × time window interaction (*F*(9,117) = 15.082, *p*<.001), (3) the pre-cueing condition × electrode interaction (*F*(12,156) = 2.623, *p* = .003) and (4) the pre-cueing condition × time window × electrode interaction (*F*(36,468) = 3.213, *p*<.001).

Independent 3-way ANOVAs (factors: pre-cueing, grip type, force) on each electrode and time windows were used to analyze in more detail how the pre-cueing effect revealed by the 5-way ANOVA varied between the different time windows and the different regions of the scalp. Only the effects with a *p*-value below the Bonferroni adjusted α (0.05/(5*4) = 0.0025) were considered. The only significant effects of pre-cueing condition occurred in the Early-PP time window for the midline electrodes Cz (*F*(3,39) = 6.805, *p*<.001), CPz (*F*(3,39) = 18.211, *p*<.001, *ε* = .48) and Pz (*F*(3,39) = 6.918, *p*<.001) and the contralateral electrode C3 (*F*(3,39) = 13.443, *p*<.001). No significant effects were observed on the ipsilateral electrode (C4).

On Cz, CPz and Pz, post-hoc analyses revealed that the ERD/ERS amplitude for the non-informative condition (NO) differed from the 3 informative conditions (ALL, GRIP, FORCE) (all *p*-values<.03). No differences were observed between the 3 informative conditions (all *p*-values>.5). Indeed, Figure 2c (Cz, CPz, Pz) shows for the Early-PP time window that the beta power stayed at a high level for the NO condition while a transient power drop occurred in the three informative conditions. The peak latency of this drop was around 500 ms on average. On C3, the following effects on beta power were observed: NO (+69 %) ≈ FORCE (+51 %) > GRIP (+6 %) ≈ ALL (+8 %) (all significant *p*<.006; all non-significant *p*>.4). Indeed, Figure 2c (C3) shows that a high level of beta power was maintained in the Early-PP time window for the NO and FORCE conditions while a transient power drop was observed for ALL and GRIP. The same ranking was observed in all individual subjects but two. One of the latter subjects showed a power drop in the FORCE at the level of the GRIP and ALL conditions. In the other subject, the drop in beta power after cue onset was absent in all conditions.

Below, this power decrease during the Early-PP time window for the 3 informative conditions on Cz, CPz, and Pz, and GRIP and ALL conditions on C3, is referred to as “PD_cue_”.

Finally, no main effects or interactions related to movement parameters (grip and force) were significant (see Figure 3 for C3). Thus, the different response types (PGHF, PGLF, SGHF, SGLF) did not differentially modulate the oscillatory activity in the beta band, neither during movement preparation, nor during the dynamic and static phases of movement execution.

## Discussion

We used EEG recordings in a pre-cueing task to assess how centro-parietal beta oscillations are modulated by the manipulation of prior information about the grip and force parameters for the planning and execution of visually-guided reach-to-grasp movements. The main findings are that beta oscillations differ between pre-cueing conditions but not between behavioral responses.

### ERD/ERS during pre-cued reach-to-grasp movements

Our data agree with previous reports on ERD/ERS modulations during motor tasks. First, we observed an ERS around the cue onset (ERS_cue_) with a fronto-central distribution [44], [45], [46]. The amplitude of ERS_cue_ was maximal over C3. Since C3 is considered to reflect mainly the activity originating from the precentral gyrus [38], our data support the hypothesis that the primary motor cortex (M1) is involved in the generation of the ERS_cue_ as shown by source localization [46] and intra-cerebral recordings [23], [47]. The ERS_cue_ started to increase before the cue onset [23], [40], [47], [48], [49]. This anticipatory power increase is absent in studies in which the visual cue could not be easily timed by the subject [27], [41], [44], but see [22]. However, despite the overall consensus about the anticipatory part of the cue-related ERS, we cannot totally exclude the possibility that the ERS_cue_ on C3 was, at least in part, a rebound elicited by the switch closure with the right hand to initiate the trial. Indeed, it has been shown that a beta rebound follows the offset of self-paced right-hand movements [50].

Second, a centro-parietal ERD emerged gradually during the PP to reach its maximum around movement onset. The pre-GO ERD is preponderant over the hemisphere contralateral to the moving hand and becomes progressively bilateral toward execution [4], [25], [41], [51]. The ERD was sustained during the dynamic phase of movement execution [52]. We probably observed such a strong pre-GO ERD since we used a fixed PP duration. Indeed, the presence of a pre-GO ERD depends on the temporal predictability of the GO onset [7], [44].

Third, following object pull, the hold period was characterized by an increase in beta power [27], [53]. This power increase is comparable to the classical beta rebound that follows movement offset [11], [21], [50], [54], [55]. In agreement with studies showing that sustained compared to brief movements elicit a weaker rebound (54,56], the beta rebound was of small amplitude in our task. The rebound amplitude over the vertex only slightly exceeded the baseline level.

### Modulations of the ERD/ERS with behavioural responses

In the present study, we observed no significant modulations of the ERD/ERS amplitude with movement parameters. In particular, we found no effects of grip type on the beta rebound, in contrast with an earlier study on monkey local field potentials (LFPs) [27]. The weak rebound that we observed may in fact characterize grasping movements requiring independent control of the thumb and fingers as for PG and SG. Spinks et al. [27] observed that in monkeys, the LFP beta rebound amplitude was weak following grasps involving the thumb in opposition to the other fingers (e.g., precision grip), while it was significantly larger for the hook grip for which the finger muscles were predominantly co-activated [57]. This interpretation is also congruent with the findings of van Elk and collaborators [53], who found a weak rebound in relation to grasping movements towards everyday-life objects for which the thumb was always involved. Stančák et al. [28] showed that the contralateral pre-movement ERD is modulated by load changes opposing self-paced index finger extensions from 30 to 80 g but not from 80 to 130 g. On the ipsilateral side, the 130 g load was accompanied by a lower ERD than the 80 g load. These discrepancies with our results may be due to differences in the experimental protocol. First, we used a higher force range (200 g and 700 g) to ensure that the subjects anticipated the force [58]. Also, our task required performing complex reach-to-grasp movements triggered by a GO signal. These movements may require the activation of additional visuomotor integrations processes in response to the GO which contrast with self-paced movements.

To conclude, despite the fact that the beta oscillations are often characterized as “sensorimotor”, our study shows that they are of limited use to differentiate (decode) between different hand positions and forces for grasping. A recent study shows that this also seems to be the case when more invasive electrocorticography (ECoG) electrodes are used for recording [30]. However, the decoding of highly differentiated grasp types might be possible on the basis of intra-cortical LFP recordings [27].

### Modulation of the ERD/ERS with the number and type of prior information

The ERS_cue_, ERD and rebound were modulated differently by the pre-cueing conditions. A significant effect of pre-cueing conditions was restricted to the Early-PP time window. In line with other studies, the beta power decreased after the cue (PD_cue_) when it contained relevant information about the forthcoming movement (except in the FORCE condition on C3, see below) but it remained unchanged in the NO condition in which the cue acted only as a temporal warning signal for the forthcoming GO [22], [23], [40], [44], [49], [59], [60], but see [41]. The PD_cue_ was transient and peaked approximately 500 ms after the cue onset [40], [44], [60]. The PD_cue_ was relatively independent of the ERS_cue_ since it was observed both when the ERS_cue_ was present as on C3 [40] or absent as on CPz. In fact, the ERS_cue_, PD_cue_, ERD and rebound were characterized by strikingly different topographies. The PD_cue_ was present on the parietal region where no ERS_cue_ was observed. The ERD was bilateral during movement execution and the rebound dominated over Cz.

Altogether, these modulations support the view that the different ERD/ERS components (ERS_cue_, PD_cue_, ERD and rebound) reflect separate processes whose respective effects on beta power may overlap in space and time. In the present study, we assume that it was especially the case on C3 after the cue onset between the ERS_cue_ and the PD_cue_. Overlap of ERP components is a common phenomenon. For instance, the early and late components of the CNV are considered to reflect separate functional processes that might overlap in space and time when a short PP(<2 s) is used [61]. Such overlap, induced by short PP duration combined with other factors such as the predictability of the cue and GO signals as well as the cue relevance, may affect the time course of some ERD/ERS components (ERS_cue_, PD_cue_, and pre-GO ERD) or even preclude their emergence. Importantly, the overlap between components means that a given functional process can occur normally even if its related beta component is altered or absent in the EEG. Altogether, the combination of experimental factors and inter-subject variability (e.g. [62]) may lead to different ERD/ERS profiles and explain discrepancies across studies. For instance, when short PPs are used, the pre-GO ERD may overlap spatio-temporally with the cue-related modulations (PD_cue_ and ERS_cue_), reducing the ERS_cue_ or altering the transient profile of the PD_cue_ by limiting the post PD_cue_ resynchronization [12], [22].

### Functional role of beta oscillations

The pre-cueing paradigm used in the present study was particularly suited to distinguish between different beta components related to the successive task events (i.e. ERS_cue_, PD_cue_, pre-GO ERD, rebound). These ERD/ERS modulations are consistent across many studies despite some variability in their spatio-temporal properties. Indeed, we have argued above that each component may be more or less salient due to possible spatio-temporal overlap between them. Our results strongly support the idea that the cortical beta activity is characterized by a “functional polymorphism” [18]. However, it remains to be understood what functional role can be attributed to each ERD/ERS component. In this section, we briefly discuss how some common functional interpretations of the beta band activity can account for our data.

#### The “attentional” hypothesis

This hypothesis links ERS over the motor cortex to attentional processes that enhance the sensitivity to relevant cues [23]. This hypothesis nicely predicts the ERS_cue_ that anticipates the cue onset. However, it does not explain why in the FORCE and NO conditions the ERS_cue_ is maintained after the cue onset when no sustained attention is required anymore [46]. Moreover, no pre-cueing effect and no ERS were observed around the GO. Such effects would have been expected since in the NO, FORCE and GRIP conditions, attention is required to extract movement-related information from the GO signal.

#### The “postural control” hypothesis

Several studies suggest that beta oscillations relate to the control of a stable posture [63], [64]. This hypothesis can explain why we obtain a rebound during the static hold relative to the preceding ERD during the dynamic phase of movement execution. However, we should have expected the ERS_cue_ to be maintained throughout the whole PP since the subjects were instructed not to move during this period of time. In addition, it remains unclear how this hypothesis can explain the pre-cueing effects observed during the early-PP.

#### The “status quo” hypothesis

Engel and Fries [17] argued that beta oscillations reflect an active process promoting the existing motor or cognitive set (i.e. the “*status quo*”). The beta power should be weaker when a change in the current motor or cognitive set is expected or intended. This hypothesis fits with the presence of an ERD preceding the changes induced by the GO signal. However, it can hardly explain why beta power peaks at the moment of the informative cue.

#### The “functional inhibition” hypothesis

It has been suggested that beta oscillations reflect a mechanism of functional inhibition of the motor cortex by somatosensory processing. The beta rebound is suppressed in the absence of tactile and proprioceptive afferences [20], [65], [66] and it can be observed in non-motor tasks such as passive movements [20], [25] and tactile stimulation [50], [67], [68]. However this hypothesis does not explain the ERS_cue_ since no particular changes in somatosensory processing occur at this stage of the task.

#### The “uncertainty” hypothesis

Tzagarakis et al. [22] showed that the pre-GO ERD above the contralateral motor cortex varies with the level of uncertainty about the direction of a forthcoming movement. In contrast, we did not observe any significant ERD difference between the ALL and NO conditions in which there was no or maximum uncertainty, respectively. However, an alternative explanation for the discripency between these two studies is that they used two types of uncertain factor: the movement direction [22] and the grip type and/or force.

To summarize, no single functional hypothesis accounts for all the beta modulations observed in our task. Importantly, the different hypotheses discussed above are not necessarily contradictory and several of them may coexist. Accordingly, it is probable that a given beta component is the resultant of the combined influence of several functional processes. It is possible to reconcile the different suggestions about the functional role of beta oscillation by considering more general hypotheses that do not link the beta rhythm or a component of this rhythm to a single function.

The “*idling*” hypothesis suggests that beta oscillations merely reflect the activation state of the sensorimotor system. An increase in the beta power reflects an “idle” state [69] while, in contrast, an ERD is linked to an activation state. This idea is supported by combined EEG/fMRI studies linking ERD to an activation of the cerebral tissue [70], [71], [72] and by intracortical recordings showing an inverse correlation between spiking activity and LFP power in the beta range [27]. However, a recent paper [73] demonstrates that LFP oscillatory power and firing rates of neurons can be dissociated in motor cortex, and that high power is associated with increased spike synchrony and phase locking of spikes to LFP oscillations, rather than an overall change in firing rates.

Finally, the “*communication*” hypothesis suggests that beta oscillations reflect long-range inter-areal communication while higher frequencies reflect intra-areal communication [74], [75]. However, this hypothesis appears controversial since there is no clear evidence that the amount of long range cortico-cortical interactions is reduced during movement execution, as could be predicted by the drop of beta power around movement onset.

Based on all these hypotheses, we tentatively propose some interpretation for the ERD/ERS modulations in the present study. These assumptions could be specifically tested in future studies. The ERS_cue_ could reflect cortico-cortical communication for an efficient movement preparation [46] and/or anticipatory increase of attention [23]. The rebound could reveal cortico-cortical communication between somatosensory and motor areas [76] for functional inhibition and/or cortico-muscular communication for postural maintenance [64] as evidenced by coherence analyses [77]. In contrast to the ERS, the ERD might reflect activity in more local networks in relation to various aspects of movement preparation, visuo-motor transformation [78] and descending motor control. Visuo-motor transformation is viewed here as a general term encompassing many processes related to stimulus identification, stimulus/response association (e.g. the grip and/or the force selection) and motor planning. The strong ERD observed during RT and MT suggests a sustained activation probably related, at least in part, to these visuo-motor processes. In the same way, visuo-motor processes are also triggered by an informative cue and may thus be indexed by a transient power decrease in the beta frequency range (PD_cue_). Interestingly, we observed a PD_cue_ in the FORCE condition over the central electrodes (Cz and CPz) but not over the contralateral electrode (C3). This spatial difference suggests that the PD_cue_ might reflect different sensory-motor processes depending on its location on the scalp. The parietal and mid-central PD_cue_ may be linked to visual and cognitive aspects of visuo-motor transformation. These processes would be triggered by the cue in all informative conditions (GRIP, FORCE and ALL) and their completion before the GO explain why the RT was shorter in the informative than the in non-informative conditions [33]. In contrast, the contralateral PD_cue_ may be more specifically related to motor planning processes that are not activated in the FORCE condition during the PP. One can hypothesize that this processes are activated only after the GO signal, which may explain the longer RT in the FORCE compared to the GRIP condition [33]. This assumption fits with the difficulty reported by all subjects to use the force cue alone to prepare the movement and confirms that the contralateral areas are more involved in low-level planning processes than the other sensorimotor regions [31], [33].

## Conclusion

Our study outlined the composite nature of the beta ERD/ERS profile. During the different task epochs, including movement preparation, execution and static holding, several beta components have been distinguished based on their temporal and spatial properties and how they are affected by the experimental factors, i.e. pre-cueing conditions and the grip and force parameters. We also propose that, in some situations, a spatio-temporal overlap between beta components may explain some discrepancies across studies related to the time course of the ERD/ERS profiles or the presence or absence of a given component. The link between oscillatory activity in the brain and cognitive function is not simple. We suggest that the beta rhythm in general and even a single component reflect many functional processes.

The present study also reveals that in a similar experimental context, the modulation of beta power shows marked differences compared with the modulation of slow ERPs like the CNV [33]. Although both the pre-GO ERD and the late CNV develops gradually during the preparation period and show approximately similar onset time, the CNV, analyzed just before the GO (late CNV), is modulated by pre-cueing condition (see [31] for a review), while the ERD is not. These observations suggest that the beta power ERD and the CNV are distinct phenomena reflecting different aspect of motor control. Crucially, we argued that the PD_cue_ may reflect local activity within the motor and/or premotor cortex dedicated to motor planning. Along with the CNV and RT modulations (discussed in [33]), the pre-cuing effect on the PD_cue_ suggests that the overall force to grasp an object cannot be planned, at least to some degree, without information about the grip type.

## References

[1] Jasper H, Penfield W (1949) Electrocorticograms in man: effect of voluntary movement upon the electrical activity of the precentral gyrus. Arch Psychiatr Zeitschr Neurol 83: 163 174.

[2] Pfurtscheller G, Lopes da Silva FH (1999) Event-related EEG/MEG synchronization and desynchronization: basic principles. Clin Neurophysiol 110: 1842–1857. 10576479 10.1016/s1388-2457(99)00141-8

[3] Salmelin R, Forss N, Knuutila J, Hari R (1995) Bilateral activation of the human somatomotor cortex by distal hand movements. Electroencephalogr Clin Neurophysiol 95: 444–452. 8536573 10.1016/0013-4694(95)00193-x

[4] Stancák Jr A, Pfurtscheller G (1996) Event-related desynchronisation of central beta-rhythms during brisk and slow self-paced finger movements of dominant and nondominant hand. Cogn Brain Res 4: 171–183. 10.1016/s0926-6410(96)00031-6 8924046

[5] Crone NE, Miglioretti DL, Gordon B, Sieracki JM, Wilson MT, et al (1998) Functional mapping of human sensorimotor cortex with electrocorticographic spectral analysis. I. Alpha and beta event-related desynchronization. Brain 121: 2271–2299. 9874480 10.1093/brain/121.12.2271

[6] Ohara S, Ikeda A, Kunida T, Yazawa S, Baba K, et al (2000) Movement-related change of electrocorticographic activity in human supplementary motor area proper. Brain 123: 1203–1215. 10825358 10.1093/brain/123.6.1203

[7] Alegre M, Gurtubay IG, Labarga A, Iriarte J, Malanda A, et al (2003) Alpha and beta oscillatory changes during stimulus-induced movement paradigms: effect of stimulus predictability. NeuroReport 14: 381–385. 12634488 10.1097/00001756-200303030-00017

[8] Sochůrková D, Rektor I, Jurák P, Stančák A (2006) Intracerebral recording of cortical activity related to self-paced voluntary movements: a Bereitschaftspotential and event-related desynchronization/synchronization. SEEG study. Exp Brain Res 173: 637–649. 16544136 10.1007/s00221-006-0407-9

[9] Szurhaj W, Derambure P, Labyt E, Cassim F, Bourriez JL, et al (2003) Basic mechanisms of central rhythms reactivity to preparation and execution of a voluntary movement: a stereoelectroencephalographic study. Clin Neurophysiol 1: 107–119. 10.1016/s1388-2457(02)00333-4 12495771

[10] Pfurtscheller G, Solis-Escalante T (2009) Could the beta rebound in the EEG be suitable to realize a “brain switch”? Clini Neurophysiol 120: 24–29. 10.1016/j.clinph.2008.09.027 19028138

[11] Gaetz W, Macdonald M, Cheyne D, Snead OC (2010) Neuromagnetic imaging of movement-related cortical oscillations in children and adults: age predicts post-movement beta rebound. Neuroimage 51: 792–807. 20116434 10.1016/j.neuroimage.2010.01.077

[12] Nakagawa K, Aokage Y, Fukuri T, Kawahara Y, Hashizume A, et al (2011) Neuromagnetic beta oscillation changes during motor imagery and motor execution of skilled movements. Neuroreport 22: 217–222. 21386697 10.1097/WNR.0b013e328344b480

[13] Salmelin R, Hari R (1994) Spatiotemporal characteristics of sensorimotor neuromagnetic rhythms related to thumb movement. Neuroscience 60: 537–550. 8072694 10.1016/0306-4522(94)90263-1

[14] Taniguchi M, Kato A, Fujita N, Hirata M, Tanaka H, et al (2000) Movement-related desynchronization of the cerebral cortex studied with spatially filtered magnetoencephalography. NeuroImage 12: 298–306. 10944412 10.1006/nimg.2000.0611

[15] Leocani L, Comi G (2006) Movement-related event-related desynchronization in neuropsychiatric disorders. Prog Brain Res 159: 351–366. 17071242 10.1016/S0079-6123(06)59023-5

[16] Devos D, Defebvre L (2006) Effect of deep brain stimulation and L-Dopa on electrocortical rhythms related to movement in Parkinson's disease. Prog Brain Res 159: 331–349. 17071241 10.1016/S0079-6123(06)59022-3

[17] Engel AK, Fries P (2010) Beta-band oscillations - signalling the status quo? Curr Opin Neurobiol 20: 156–165. 20359884 10.1016/j.conb.2010.02.015

[18] Jenkinson N, Brown P (2011) New insight into the relationship between dopamine, beta oscillations and motor function. Trends Neurosci 34: 611–618. 22018805 10.1016/j.tins.2011.09.003

[19] Feige B, Kristeva-Feige R, Rossi S, Pizzella V, Rossini PM (1996) Neuromagnetic study of movement-related changes in rhythmic brain activity. Brain Res 734: 252–260. 8896832

[20] Cassim F, Monaca C, Szurhaj W, Bourriez JL, Defebvre L, et al (2001) Does post-movement beta synchronization reflect an idling motor cortex? Neuroreport 12: 3859–3863. 11726809 10.1097/00001756-200112040-00051

[21] Jurkiewicz MT, Gaetz WC, Bostan AC, Cheyne D (2006) Post-movement beta rebound is generated in motor cortex: evidence from neuromagnetic recordings. Neuroimage 32: 1281–1289. 16863693 10.1016/j.neuroimage.2006.06.005

[22] Tzagarakis C, Ince NF, Leuthold AC, Pellizzer G (2010) Beta-band activity during motor planning reflects response uncertainty. J Neurosci 30: 11270–11277. 20739547 10.1523/JNEUROSCI.6026-09.2010 PMC6633326

[23] Saleh M, Reimer J, Penn R, Ojakangas CL, Hatsopoulos NG (2010) Fast and slow oscillations in human primary motor cortex predict oncoming behaviorally relevant cues. Neuron 65: 461–471. 20188651 10.1016/j.neuron.2010.02.001 PMC3199221

[24] Neuper C, Pfurtscheller G (1999) Motor imagery and ERD. In: Pfurtscheller G, Lopes da Silva FH, eds. Event-related desynchronization. Handbook of electroencephalography and clinical neurophysiology. Amsterdam: Elsevier. pp 303–325.

[25] Müller GR, Neuper C, Rupp R, Keinrath C, Gerner HJ, et al (2003) Event-related beta EEG changes during wrist movements induced by functional electrical stimulation of forearm muscles in man. Neurosci Lett 340: 143–147. 12668257 10.1016/s0304-3940(03)00019-3

[26] Koelewijn T, van Schie HT, Bekkering H, Oostenveld R, Jensen O (2008) Motor-cortical beta oscillations are modulated by correctness of observed action. Neuroimage 40: 767–775. 18234516 10.1016/j.neuroimage.2007.12.018

[27] Spinks RL, Kraskov A, Brochier T, Umilta MA, Lemon RN (2008) Selectivity for grasp in local field potential and single neuron activity recorded simultaneously from M1 and F5 in the awake macaque monkey. J Neurosci 28: 10961–10971. 18945904 10.1523/JNEUROSCI.1956-08.2008 PMC2637078

[28] Stancák Jr A, Riml A, Pfurtscheller G (1997) The effects of external load on movement-related changes of the sensorimotor EEG rhythms. Electroencephalogr Clin Neurophysiol 102: 495–504. 9216482 10.1016/s0013-4694(96)96623-0

[29] Rickert J, Oliveira SC, Vaadia E, Aertsen A, Rotter S, et al (2005) Encoding of movement direction in different frequency ranges of motor cortical local field potentials. J Neurosci 25: 8815–8824. 16192371 10.1523/JNEUROSCI.0816-05.2005 PMC6725584

[30] Pistohl T, Schulze-Bonhage A, Aertsen A, Mehring C, Ball T (2012) Decoding natural grasp types from human ECoG. Neuroimage 59: 248–260. 21763434 10.1016/j.neuroimage.2011.06.084

[31] Leuthold H, Sommer W, Ulrich R (2004) Preparing for Action: Inferences from CNV and LRP. J Psychophysiol 18: 77–88.

[32] Rosenbaum DA (1980) Human movement initiation: specification of arm, direction, and extent. J Exp Psychol Gen 109: 444–474. 6449531 10.1037//0096-3445.109.4.444

[33] Zaepffel M, Brochier T (2012) Planning of visually-guided reach-to-grasp movements: inference from reaction time and contingent negative variation (CNV). Psychophysiology 49: 17–30. 21895686 10.1111/j.1469-8986.2011.01277.x

[34] Kayser J, Tenke CE (2006) Principal components analysis of Laplacian waveforms as a generic method for identifying ERP generator patterns: I. Evaluation with auditory oddball tasks. Clini Neurophysiol 117: 348–368. 10.1016/j.clinph.2005.08.034 16356767

[35] Perrin F, Pernier J, Bertrand O, Echallier JF (1989) Spherical splines for scalp potential and current density mapping. Electroencephalogr Clin Neurophysiol 72: 184–187. 2464490 10.1016/0013-4694(89)90180-6

[36] Garcia S, Fourcaud-Trocmé N (2009) OpenElectrophy: an electrophysiological data- and analysis-sharing framework. Front Neuroinform 3: 14 doi:10.3389/neuro.11.014.2009 19521545 10.3389/neuro.11.014.2009 PMC2694696

[37] Roux SG, Cenier T, Garcia S, Litaudon P, Buonviso N (2007) A wavelet-based method for local phase extraction from a multi-frequency oscillatory signal. J Neurosci Methods 160: 135–143. 17049617 10.1016/j.jneumeth.2006.09.001

[38] Homan RW, Herman J, Purdy P (1987) Cerebral location of international 10-20 system electrode placement. Electroencephalogr Clini Neurophysiol 66: 376–382. 10.1016/0013-4694(87)90206-9 2435517

[39] Deiber MP, Ibañez V, Sadato N, Hallett M (1996) Cerebral structures participating in motor preparation in humans: a positron emission tomography study. J Neurophysiol 75: 233–247. 8822554 10.1152/jn.1996.75.1.233

[40] van Wijk BCM, Daffertshofer A, Roach N, Praamstra P (2009) A role of beta oscillatory synchrony in biasing response competition. Cereb Cortex 19: 1294–1302. 18836098 10.1093/cercor/bhn174

[41] Doyle LMF, Yarrow K, Brown P (2005) Lateralization of event-related beta desychronization in the EEG during pre-cued reaction time tasks. Clin Neurophysiol 116: 1879–1888. 15979401 10.1016/j.clinph.2005.03.017

[42] Vecchiato G, De Vico Fallani F, Astolfi L, Toppi J, Cincotti F, et al (2010) The issue of multiple univariate comparisons in the context of neuroelectric brain mapping: An application in a neuromarketing experiment. J Neurosci Methods 191: 283–289. 20637802 10.1016/j.jneumeth.2010.07.009

[43] Greenhouse WW, Geisser S (1959) On methods in the analysis of profile data. Psychometrika 24: 95–112.

[44] Alegre M, Imirizaldu L, Valencia M, Iriarte J, Arcocha J, et al (2006) Alpha and beta changes in cortical oscillatory activity in a go/no go randomly-delayed-response choice reaction time paradigm. Clin Neurophysiol 117: 16–25. 16316781 10.1016/j.clinph.2005.08.030

[45] Molnár M, Csuhaj R, Gaál ZA, Czigler B, Ulbert I, et al (2008) Spectral characteristics and linear-nonlinear synchronization changes of different EEG frequency bands during the CNV. Psychophysiology 45: 412–419. 18266804 10.1111/j.1469-8986.2008.00648.x

[46] Fischer T, Langner R, Diers K, Brocke B, Birbaumer N (2010) Temporo-spatial dynamics of event-related EEG beta activity during the initial contingent negative variation. PloS One 5:pii: e12514. 20824080 10.1371/journal.pone.0012514 PMC2932695

[47] Takahashi K, Saleh M, Penn RD, Hatsopoulos N (2011) Propagating waves in human motor cortex. Front Hum Neurosci 5: 40 doi:10.3389/fnhum.2011.00040 21629859 10.3389/fnhum.2011.00040 PMC3084448

[48] Kilavik BE, Riehle A (2010) Timing structures neuronal activity during preparation for action. In: Nobre AC, Coull JT, eds. Attention and time. Oxford: Oxford University Press. pp 257–271.

[49] Kilavik BE, Ponce-Alvarez A, Trachel R, Confais J, Takerkart S, et al (2012) Context-Related Frequency Modulations of Macaque Motor Cortical LFP Beta Oscillations. Cerebral Cortex 22: 2148–59. 22021914 10.1093/cercor/bhr299

[50] Neuper C, Pfurtscheller G (2001) Evidence for distinct beta resonance frequencies in human EEG related to specific sensorimotor cortical areas. Clin Neurophysiol 112: 2084–2097. 11682347 10.1016/s1388-2457(01)00661-7

[51] Rau C, Plewnia C, Hummel F, Gerloff C (2003) Event-related desynchronization and excitability of the ipsilateral motor cortex during simple self-paced finger movements. Clin Neurophysiol 114: 1819–1826. 14499743 10.1016/s1388-2457(03)00174-3

[52] Erbil N, Ungan P (2007) Changes in the alpha and beta amplitudes of the central EEG during the onset, continuation, and offset of long-duration repetitive hand movements. Brain Res 1169: 44–56. 17689502 10.1016/j.brainres.2007.07.014

[53] van Elk M, van Schie HT, van den Heuvel R, Bekkering H (2010) Semantics in the motor system: motor-cortical beta oscillations reflect semantic knowledge of end-postures for object use. Front Hum Neurosci 4: 8 doi:10.3389/neuro.09.008.2010 20161997 10.3389/neuro.09.008.2010 PMC2821178

[54] Alegre M, Labarga A, Gurtubay IG, Iriarte J, Malanda A, et al (2003) Movement-related changes in cortical oscillatory activity in ballistic sustained and negative movements. Exp Brain Res 148: 17–25. 12478393 10.1007/s00221-002-1255-x

[55] Alegre M, Alvarez-Gerriko I, Valencia M, Iriarte J, Artieda J (2008) Oscillatory changes related to the forced termination of a movement. Clin Neurophysiol 119: 290–300. 18083620 10.1016/j.clinph.2007.10.017

[56] Cassim F, Szurhaj W, Sediri H, Devos D, Bourriez J, et al (2000) Brief and sustained movements: differences in event-related (de)synchronization (ERD/ERS) patterns. Clin Neurophysiol 111: 2032–2039. 11068239 10.1016/s1388-2457(00)00455-7

[57] Brochier T, Spinks RL, Umilta MA, Lemon RN (2004) Patterns of muscle activity underlying object-specific grasp by the macaque monkey. J Neurophysiol 92: 1770–1782. 15163676 10.1152/jn.00976.2003

[58] Gordon AM, Westling G, Cole KJ, Johansson RS (1993) Memory representations underlying motor commands used during manipulation of common and novel objects. J Neurophysiol 69: 1789–96. 8350123 10.1152/jn.1993.69.6.1789

[59] Wheaton L, Fridman E, Bohlhalter S, Vorbach S, Hallett M (2009) Left parietal activation related to planning, executing and suppressing praxis hand movements. Clin Neurophysiol 120: 980–986. 19345141 10.1016/j.clinph.2009.02.161 PMC2680923

[60] Pastötter B, Berchtold F, Bäuml KHT (2011) Oscillatory correlates of controlled speed-accuracy tradeoff in a response-conflict task. Hum Brain Mapp doi:101002/hbm.21322 10.1002/hbm.21322 PMC6869900 21618665

[61] Rohrbaugh J, Gaillard AWK (1983) Sensory and motor aspects of the contingent negative variation. In: Gaillard AWK, Ritter W, eds. Tutorials in event-related potential research: Endogenous components. \Amsterdam: Elsevier. pp 269–310.

[62] Müller-Gerking J, Pfurtscheller G, Flyvbjerg H (2000) Classification of movement-related EEG in a memorized delay task experiment. Clin Neurophysiol 111: 1353–1365. 10904215 10.1016/s1388-2457(00)00345-x

[63] Baker SN, Kilner JM, Pinches EM, Lemon RN (1999) The role of synchrony and oscillation in the motor output. Exp Brain Res 128: 109–117. 10473748 10.1007/s002210050825

[64] Kristeva R, Patino L, Omlor W (2007) Beta-range cortical motor spectral power and corticomuscular coherence as a mechanism for effective corticospinal interaction during steady-state motor output. Neuroimage 36: 785–792. 17493837 10.1016/j.neuroimage.2007.03.025

[65] Houdayer E, Labyt E, Cassim F, Bourriez JL, Derambure P (2006) Relationship between event-related beta synchronization and afferent inputs: analysis of finger movement and peripheral nerve stimulations. Clini Neurophysiol 117: 628–636. 10.1016/j.clinph.2005.12.001 16427358

[66] Reyns N, Houdayer E, Bourriez JL, Blond S, Derambure P (2008) Post-movement beta synchronization in subjects presenting with sensory deafferentation. Clin Neurophysiol 119: 1335–1345. 18417418 10.1016/j.clinph.2008.02.020

[67] Stancák Jr A, Svoboda J, Rachmanova R, Vrana J, Kralik J, et al (2003) Desynchronization of cortical rhythms following cutaneous stimulation: effects of stimulus repetition and intensity, and of the size of corpus callosum. Clin Neurophysiol 114: 1936–1947. 14499756 10.1016/s1388-2457(03)00201-3

[68] Gaetz W, Cheyne D (2006) Localization of sensorimotor cortical rhythms induced by tactile stimulation using spatially filtered MEG. Neuroimage 30: 899–908. 16326116 10.1016/j.neuroimage.2005.10.009

[69] Pfurtscheller G, Stancák Jr A, Neuper C (1996) Post-movement beta synchronization. A correlate of an idling motor area? Electroencephalogr Clin Neurophysiol 98: 281–293. 8641150 10.1016/0013-4694(95)00258-8

[70] Formaggio E, Storti SF, Avesani M, Cerini R, Milanese F, et al (2008) EEG and FMRI coregistration to investigate the cortical oscillatory activities during finger movement. Brain Topogr 21: 100–111. 18648924 10.1007/s10548-008-0058-1

[71] Yuan H, Liu T, Szarkowski R, Rios C, Ashe J, et al (2010) Negative covariation between task-related responses in alpha/beta-band activity and BOLD in human sensorimotor cortex: an EEG and fMRI study of motor imagery and movements. Neuroimage 49: 2596–2606. 19850134 10.1016/j.neuroimage.2009.10.028 PMC2818527

[72] Stevenson CM, Brookes MJ, Morris PG (2011) β-Band correlates of the fMRI BOLD response. Hum Brain Mapp 32: 182–197. 21229612 10.1002/hbm.21016 PMC6870318

[73] Engelhard B, Ozeri N, Israel Z, Bergman H, Vaadia E (2013) Inducing gamma oscillations and precise spike synchrony by operant conditioning via brain-machine interface. Neuron 77: 361–375. 23352171 10.1016/j.neuron.2012.11.015

[74] Kopell N, Ermentrout GB, Whittington MA, Traub RD (2000) Gamma rhythms and beta rhythms have different synchronization properties. Proc Natl Acad Sci U S A 97: 1867–1872. 10677548 10.1073/pnas.97.4.1867 PMC26528

[75] von Stein A, Sarnthein J (2000) Different frequencies for different scales of cortical integration: from local gamma to long range alpha/theta synchronization. Int J Psychophysiol 38: 301–313. 11102669 10.1016/s0167-8760(00)00172-0

[76] Brovelli A, Ding M, Ledberg A, Chen Y, Nakamura R, et al (2004) Beta oscillations in a large-scale sensorimotor cortical network: directional influences revealed by Granger causality. Proc Natl Acad Sci USA 101: 9849–9854. 15210971 10.1073/pnas.0308538101 PMC470781

[77] Baker S (2007) Oscillatory interactions between sensorimotor cortex and the periphery. Curr Opin Neurobiol 17: 649–655. 18339546 10.1016/j.conb.2008.01.007 PMC2428102

[78] Grol MJ, Majdandžić J, Stephan KE, Verhagen L, Dijkerman HC, et al (2007) Parieto-frontal connectivity during visually guided grasping. J Neurosci 27: 11877–11887. 17978028 10.1523/JNEUROSCI.3923-07.2007 PMC2703728

